# Long-Term Effect of COVID-19 on Lung Imaging and Function, Cardiorespiratory Symptoms, Fatigue, Exercise Capacity, and Functional Capacity in Children and Adolescents: A Systematic Review and Meta-Analysis

**DOI:** 10.3390/healthcare10122492

**Published:** 2022-12-09

**Authors:** Catherine Campos, Samantha Prokopich, Hal Loewen, Diana C. Sanchez-Ramirez

**Affiliations:** 1 Department of Respiratory Therapy, College of Rehabilitation Sciences, University of Manitoba, Winnipeg, MB R3T 2N2, Canada; 2 Neil John Maclean Health Sciences Library, University of Manitoba, Winnipeg, MB R3T 2N2, Canada

**Keywords:** COVID-19, lung imaging and function, cardiorespiratory symptoms, children

## Abstract

Background: The long-term sequela of COVID-19 on young people is still unknown. This systematic review explored the effect of COVID-19 on lung imaging and function, cardiorespiratory symptoms, fatigue, exercise capacity and functional capacity in children and adolescents ≥ 3 months after infection. Methods: A systemic search was completed in the electronic databases of PubMed, Web of Science and Ovid MEDLINE on 27 May 2022. Data on the proportion of participants who had long-term effects were collected, and one-group meta-analysis were used to estimate the pooled prevalence of the outcomes studied. Results: 17 articles met the inclusion criteria, presented data on 124,568 children and adolescents. The pooled prevalence of abnormalities in lung imaging was 10% (95% CI 1–19, I^2^ = 73%), abnormal pulmonary function was 24% (95% CI 4–43, I^2^ = 90%), chest pain/tightness was 6% (95% CI 3–8, I^2^ = 100%), heart rhythm disturbances/palpitations was 6% (95% CI 4–7, I^2^ = 98%), dyspnea/breathing problems was 16% (95% CI 14–19, I^2^ = 99%), and fatigue was 24% (95% CI 20–27, I^2^ = 100%). Decreased exercise capacity and functional limitations were found in 20% (95% CI 4–37, I^2^ = 88%) and 48% (95% CI 25–70, I^2^ = 91%) of the participants studied, respectively. Conclusion: Children and adolescents may have persistent abnormalities in lung imaging and function, cardiorespiratory symptoms, fatigue, and decreased functional capacity between 3 to 12 months after infection. More research is needed to understand the long-term effect of COVID-19 on young people, and to clarify its causes and effective management.

## 1. Background

The Coronavirus disease 2019 (COVID-19), caused by the severe acute respiratory syndrome Coronavirus 2 (SARS-CoV-2) pathogen, has resulted in approximately 615 million confirmed cases of SARS-CoV-2 and 6.5 million deaths worldwide as of 3 October 2022 [1]. Evidence largely suggests that the novel coronavirus can cause lasting consequences after the acute phase of the infection in COVID-19 survivors [2]. The World Health Organization (WHO) defined the long-term sequela of COVID-19, called long COVID, as a condition characterised by the presence of persistent COVID-19 symptoms in individuals with a history of probable or confirmed SARS-CoV-2 infection, lasting beyond 3 months of the acute phase of the infection, that cannot be explained by an alternative diagnosis [3].

A high prevalence of persistent chest imaging and in pulmonary function tests abnormalities along with persistent symptoms, decreased functional capacity and decreased quality of life have been reported in adult populations several months after COVID-19 infection [4,5]. Some of the identified contributing risk factors towards the development of long COVID include female sex, increased age, presence of comorbidities, and severe COVID-19 infection (e.g., abnormal auscultation findings, symptom severity, ICU admission and oxygen therapy) [6,7,8,9,10].

Although children tend to experience mild or no symptoms during the acute phase of COVID-19, several studies have reported persistent symptoms such as fatigue, cough, dyspnea, and decreased functional capacity in children and adolescents up to 12 weeks post-infection [11,12,13,14]. Evidence suggests that long COVID symptoms can resolve in most children within months of the initial infection, [11], however, there is limited information about the impact of long COVID in this group of patients beyond the 3 months. The aim of this systematic review was to explore the persistent effects of COVID-19 beyond 3 months post-infection on children and adolescents’ lung imaging and function, cardiorespiratory symptoms, fatigue, exercise capacity, and functional limitations.

## 2. Methods

### 2.1. Protocol and Registration

This systematic review, registered in the International Prospective Register for Systematic Reviews (PROSPERO) (CRD42022327478), was conducted using the Preferred Reporting Items for Systematic Reviews and Meta-Analyses (PRISMA) [15].

### 2.2. Literature Search and Study Selection

A search was conducted in the electronic databases of PubMed, Web of Science and Ovid MEDLINE on 27 May 2022 using the strategy and key words contained in Supplementary Table S1. The reference lists of the chosen studies were reviewed to retrieve relevant publications that were not found in the initial search. Inclusion criteria included: (1) reporting the effect of COVID-19 in children and/or adolescents regarding at least one of these outcomes: lung imaging (chest computerized tomography (CT) scan or X-ray), lung function, cardiorespiratory symptoms, fatigue, exercise capacity, and functional limitations (activities of daily living); (2) the follow-up time was ≥3 months; (3) the publication contained primary data and was not a systematic review or meta-analysis. Two reviewers (SP and CC) used the Quality Assessment tool recommended by the National Heart, Lung, and Blood Institute (NHLBI) to assess the quality of the cross-sectional and observational cohort studies that were selected [16].

**Figure 1:**
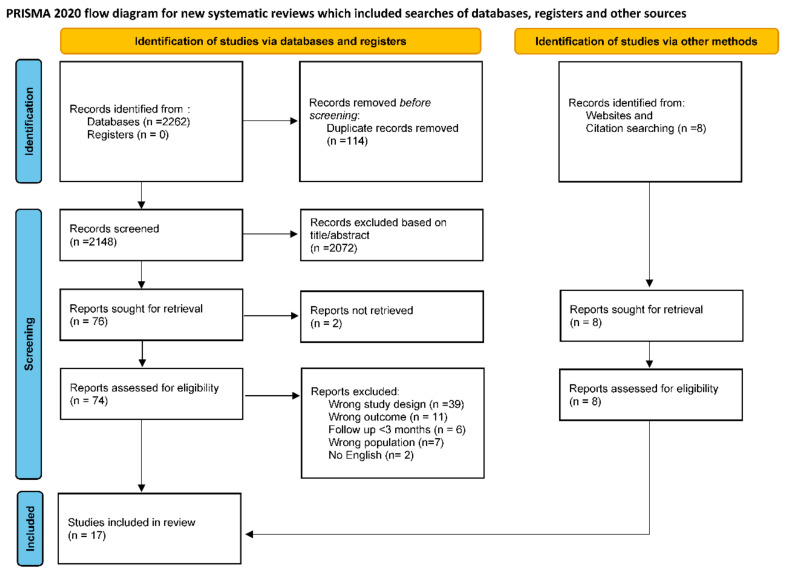
Diagram flow of studies screened and included in the review and meta-analysis.

The systematic search identified 2262 publications, and 8 additional publications were retrieved from other sources, including citation search and websites. After removing duplicates, two researchers (SP and CC) independently screened 2156 titles and abstracts, then read 84 full texts. 17 articles were selected that met the inclusion criteria (Figure 1). Primary reasons for exclusion of the studies included: (1) the publication presented data from post-COVID adults or children with specific comorbidities (e.g., multisystemic inflammatory syndrome, asthma, diabetes, etc.); (2) the publication’s follow-up time was less than 3 months aster infection; (3) the study did not present primary data (e.g., opinion, protocol, etc.); (4) the article did not present the effect of COVID-19 on any of the inclusion outcomes; and (5) the full text of the article was unavailable in English.

### 2.3. Data Extraction and Synthesis

The main characteristics of the studies such as (1) author(s)’ name(s); (2) year of publication; (3) country of the study; (4) study design; (5) follow-up time after the COVID-19 infection; (6) description of the post-COVID-19 population studied (e.g., number of participants, age, percentage of male, percentage with severe disease at baseline, and percentage of participants with long COVID) were extracted.

Data on abnormal lung imaging (CT or X-rays), abnormal lung function (pulmonary function tests- PFTs), presence of cardiorespiratory symptoms (chest pain/tightness, heart rhythm disturbances/palpitations, dyspnea/breathing difficulties, cough, rhinorrhea, sore throat), fatigue, decreased exercise capacity, and decreased functional capacity were gathered from the identified studies. Pooled prevalence was calculated using Review Manager (RevMan; version 5.3; Copenhagen, Denmark) software. Analyses were displayed as the prevalence based on the outcomes’ presented by using one-group meta-analysis, and random-effects methods The I^2^ statistic was used to present between-study heterogeneity, as the authors used the I^2^ index to describe the percentage of variation caused by inconsistency rather than chance between studies in the pooled analyses. Low, moderate, and considerable heterogeneity were indicated by I^2^ ≤ 30%, between 30–50%, and ≥ 75%, respectively [17].

## 3. Results

In this review, 17 articles reported the lasting effects of COVID-19 on children and adolescents’ lung imaging and lung function, cardiorespiratory symptoms, fatigue, exercise capacity, and activity limitations beyond 3-months follow-up [18,19,20,21,22,23,24,25,26,27,28,29,30,31,32,33,34]. The studies were completed in the following countries: Denmark (2), Australia (1), Brazil (1), Czech Republic (1), England (1), Germany (1), Iran (1), Israel (1), Italy (1), Netherlands (1), Russia (1), Switzerland (1), Turkey (1), and USA (1), while 2 studies were multinational [24,28]. Persistent outcomes were reported in 124,568 of post-COVID-19 children and adolescents between 3 to 12 months after symptom onset or hospital discharge. The participants studied had an age range between 0 and 19 years, 48% were male. Two studies focused on younger participants, with a median age of 3 years [28,33]. Severe or critical COVID-19 illness caused at baseline, defined as the need for hospitalization, ICU admission, and use of oxygen therapy was reported in approximately 1.5% of patients. Additional information describing the selected publications is shown in Table 1. Five of the studies [19,22,24,25,29] included the presence of long-COVID symptoms as an inclusion criterion for participation in the study. The mean prevalence of long COVID based on the remaining 12 studies was 30% (SD 19) [18,20,21,23,26,27,28,30,31,32,33,34]. Female sex, older age, multiple symptoms during acute infection, longer hospital stays, and respiratory distress following acute COVID-19 were risk factors contributing to long COVID in children and adolescents [18,28].

Based on the NHLBI’ Quality Assessment Tool for Observational Cohort and Cross-Sectional Studies: one study was rated as “good”, thirteen studies as “fair”, and three as “poor” (Supplementary Table S2). Table 2 presents pooled prevalence of persistent COVID-19 effects in chest imaging, lung function, cardiorespiratory symptoms, fatigue, exercise capacity, and functional capacity after 3 to 12 months o infection.

### 3.1. Lung Imaging and Lung Function (Supplementary Table S3)

Four articles reported the effect of COVID-19 infection on chest CT scans and/or X-rays in children and adolescents after 3 months follow-up [19,25,27,29]. Pooled analysis indicated that CT and X-ray abnormalities were present in 10% (95% CI 1–19, I^2^ = 73%) of 185 participants (Table 2). Three studies [19,25,31] reported abnormal PFT function in 24% (95% CI 4–43, I^2^ = 90%) of 149 pooled participants. Abnormal PFT values included abnormal spirometry results (FEV1 < 80% or FEV1/FVC < 0.8), positive bronchodilator response (ΔFEV1 ≥ 12%), or abnormal plethysmography (RV/TLC > 125%). 7% of 139 participants (CI 3–12, I^2^ = 0%) experienced an obstructive pattern [19,29,31], and 5% of 129 patients (95% CI 0–10, I^2^ = 42%) had an impaired diffusion pattern at follow-up [19,25,31] (Table 2). One study [31] revealed that one out of 50 patients (2%) experienced a restrictive pattern 3 months after hospital discharge.

### 3.2. Cardiorespiratory Symptoms and Fatigue (Supplementary Table S4)

12 articles [19,20,21,22,23,24,25,26,30,31,32,34] reported persistent cardiac symptoms in young people beyond 3 months post infection. Pooled analysis indicated that chest pain/tightness were reported in 6% of 11 135 participants (95% CI 3–8, I^2^ = 100%) [19,20,22,23,25,26,30,31,32,34] and 6% of 15 414 participants (95% CI 4–7, I^2^ = 99%) had heart rhythm disturbances/palpitations at follow-up [19,20,21,22,23,24] (Table 2). Studies in which post-COVID symptoms were not considered a requirement for inclusion showed lower prevalence of cardiac symptoms [20,21,23].

Seventeen studies investigated persistent respiratory symptoms [18,19,20,21,22,23,24,25,26,27,28,29,30,31,32,33,34] and 14 studies explored fatigue [18,19,20,21,22,23,24,26,28,29,30,32,33,34] resulting from COVID-19 infection (Supplementary Table S4). The most commonly reported respiratory symptom was dyspnea and breathing problems in 16% of 22,084 participants (95% CI 14–19, I^2^ = 99%), followed by sore throat in 10% of 19 596 patients (95% CI 8–13, I^2^ = 99%), persistent cough in 4% of 22 708 participants (95% CI 3–5, I^2^ = 98%), and rhinorrhea in 2% of 2596 patients (95% CI 0–4, I^2^ = 83%) (Table 2). Pooled analysis showed that 24% of 22 721 participants experienced prolonged fatigue (95% CI 20–27. I^2^ = 100%) 3 months beyond COVID-19 infection (Table 2). Post-exertional malaise was reported in one study, where 45% of 428 participants experienced this symptom at a mean follow-up of 8.2 months [19].

### 3.3. Exercise Capacity and Functional Limitations (Supplementary Table S5)

Decreased exercise capacity or limitations in activities of daily living were reported in 5 studies [18,19,22,24,25,29]. Decreased exercise capacity was observed in 20% of 126 participants (95% CI 4–37, I^2^ = 88%) (Table 2). Exercise intolerance was assessed using surveys [18], chart reviews and the 6 min walk test (6MWT) [25,29]. 48% of 148 participants (95% CI 25–70, I^2^ = 91%) had functional limitations including the inability or limited ability to perform activities of daily living (ADL) such as going to school [19,22] (Table 2).

## 4. Discussion

The results of this systematic review of the literature and meta-analysis reveal that children and adolescents may experience persistent lung imaging changes, lung function abnormalities, cardiorespiratory symptoms, fatigue, decreased exercise capacity and functional limitations beyond 3 months after COVID-19 infection. The most commonly reported symptoms included fatigue and dyspnea. Exercise limitations were identified in 1 in 5 studies, and functional limitations were reported in almost half of the children and adolescents studied. The lingering effects of COVID-19 identified in youth are similar to those in adults, but less prevalent (5).

Findings of post COVID-19 CT chest scans or X-rays were reported in 4 studies, which identified persistent abnormalities in 10% (n = 21) of the patients studied. It is important to note that CT and X-ray abnormalities within the paediatric population may be underrepresented due to limiting factors such as radiation safety regulations [35]. It was found that cases of pleural effusion, hyperinflation, perihilar opacities, and basal adhesions cleared 4 to 7 months after COVID-19 diagnosis [19,25,27,29]. Evidence indicated that CT scan abnormalities are commonly linked to severe acute COVID-19 disease, and radiological improvements are frequently seen in the majority of patients at 1-year follow-up [36].

There is limited evidence that persistent lung changes such as CT abnormalities are associated with respiratory symptoms in post COVID-19 patients, this is mainly due to the presence of breathlessness without imaging abnormalities. A recent study identified impaired gas exchange in the lungs of post-COVID-19 patients with normal chest CT scans who experienced dyspnea [37]. Abnormal pulmonary function, impaired diffusion capacity for carbon monoxide (DLCO) and an obstructive patter were identified in 24%, 5% and 7% of the children and adolescents studied. DLCO is used as a marker to determine pulmonary vascular diseases. A lowered DLCO indicates pulmonary diffusion abnormalities in the airways such as pulmonary fibrosis, emphysema or pulmonary emboli [38]. It has been suggested that pulmonary fibrosis may persist in post COVID-19 patients [39]. However, literature regarding the prevalence of pulmonary fibrosis in paediatric populations is limited [40,41]. During spirometry, there was a high response rate of over 50% to standardized bronchodilation tests that administer salbutamol, a common drug used to alleviate inflammation of the airways [19,25]. The high response rate to bronchodilator treatment in children demonstrates reversibility in obstructive lung patterns and may be due to the increasing prevalence of bronchial asthma in the general paediatric population, along with the unknown link between asthma and long COVID in the current literature [25].

Although long COVID is primarily considered a respiratory tract infection, it is well-documented as a multisystem disease in both adults and children that can adversely affect other organs [42,43]. However, the effects of long COVID on the cardiovascular system remains unclear [44]. It has been primarily suggested that a condition termed multisystem inflammatory syndrome in children (MIS-C) has been temporarily associated with the post-acute phase of COVID-19 [45]. MIS-C can occur in asymptomatic children and is characterized by disturbances in cardiac, respiratory, gastrointestinal and neurological symptoms [46]. A smaller study conducted by Aziz et al. [47], found that cardiac symptoms such as coronary abnormalities, pericardial effusion, and impaired left ventricle function were found in 36.5% of children with MIS-C during acute infection, but most cardiac symptoms resolved within 1 to 3 months after diagnosis.

Additional long COVID-19 symptoms identified in this review, such as heart rhythm disturbances, chest pain/tightness, persistent fatigue, and decreased exercise capacity, are commonly reported in patient’s experiencing dysautonomia, a medical condition of autonomic dysregulation that can be triggered by various viruses [48]. The diagnosis and management of this condition still needs further study.

Female sex, older age, multiple symptoms during acute COVID-19 infection, longer hospital stays, and respiratory distress following acute COVID-19 were risk factors related to long COVID in children and adolescents [18,28]. Experiencing severe symptoms during the acute phase of infection, being unvaccinated for COVID-19, and a high body mass index (BMI) that is equal to or greater than the 85th percentile for age and sex, can also increase the risk of long-COVID in this group of patients [49]. Maddux et al. [50] reported that patients under 21 years of age were more likely to experience long COVID symptoms at 2 to 4 months follow-up if they had pre-existing respiratory conditions or co-morbidities including MIS-C. The authors also found a correlation between obesity and persistent activity impairment in long COVID [50].

### 4.1. Implications and Considerations 

This study helps to fill a significant knowledge gap within the literature about the ongoing effects of COVID-19 on children and adolescents beyond 3 months post infection. Little is known about long COVID manifestations in younger populations, thus, investigating this can potentially help understand the impact of long COVID in this group of patients. Recognizing the impact of long COVID in children and adolescents can also facilitate early diagnosis and better management of this condition, which can have a major impact on the patient’s quality of life. Outcomes of this study may also help to acknowledge the ongoing need for programs and additional resources targeted to the needs of this group of patients.

### 4.2. Study Limitations

It is important to consider some factors when interpreting the results of this paper. First, there is uncertainty about the presence of the outcomes studied prior to COVID-19 infection, raising the possibility that patients may have had them prior to COVID-19 diagnosis. Therefore, other potential causes of persistent symptoms such as pre-existing respiratory conditions should also be considered when diagnosing long COVID. Secondly, heterogeneity in the studies regarding participants selection, outcomes definition, data collection tools and interpretation, different follow-up periods, and the variation in interpretation of survey questions (e.g., patient, caregiver, or healthcare provider) may influence the generalizability of results in this study. Nevertheless, the authors believe that this study will help fill a knowledge gap regarding long-COVID in children and adolescents lasting beyond 3 months after acute infection.

## 5. Conclusions

The results indicate that, similar to adults, children and adolescents suffering from long COVID may experience persistent abnormalities in lung imaging and function, cardiorespiratory symptoms, fatigue, decreased exercise capacity and decreased functional capacity between 3 to 12 months after the infection. Fatigue and dyspnea were among the most commonly reported symptoms associated with long COVID. Further studies are needed to determine the effects of COVID-19 on children and adolescents beyond 12 months, which may help understand the impact, increase awareness, and facilitate early diagnosis and potential management of this condition. Outcomes of this study may also help to acknowledge the ongoing need for programs and additional resources targeted to the needs of this group of patients.

## Figures and Tables

**Table 1:** Characteristics of the studies included (n = 17).

Author Year	Country	Study Design	Follow-Up Time	Participants				
				N	Age (Years) Mean	Male (%)	Severe Cases at Baseline n (%)	Long COVID-19 (%)
Asadi-Pooya, A. et al., 2021 [18]	Iran	Cross-sectional ^¥^	≥3 months after infection	58	12 (SD 3.3) range 6–17	48%	58 (100%) hospitalized 10 (17%) ICU	45%
Ashkenazi-Hoffnung, L. et al., 2021 [19]	Israel	Prospective	Median 112 (IQR 33–410) days after COVID-19 diagnosis	90	12 (SD 5.0) range 0–18 years	59%	11 (12%) hospitalized	100% *
Brackel, H. et al., 2021 [22]	Netherlands	Cross-sectional ^¥^	≥3 months after COVID-19 infection	89	Median 13 (IQR 9–15) range 2–18	NR	NR	100% *
Buonsenso, D. et al., 2021 [23]	Italy	Cross-sectional ^¥^	Mean 163 (SD 113.7) days after COVID-19 diagnosis	129	11 (SD 4.4)	52%	6 (5%) hospitalized 3 (2%) ICU	56%
Buonsenso, D. et al., 2022 [24]	United Kingdom, USA, and other	Cross-sectional ^¥^	Mean 8.2 (SD 3.9) months after COVID-19	510	10 (SD 3.8) (IQR 8–13) range 1–18 years	44%	22 (4%) hospitalized	100% *
Dolezalova, K. et al., 2022 [25]	Czech Republic	Prospective observational	3–6 months after COVID-19 diagnosis (5 patients: 3 ≤ 6 months and 34 patients 6 months)	39	Median 14 (IQR 8–15) range 2–18 years	44%	NA	100% *
Erol, N. et al., 2021 [26]	Turkey	Case control	Mean 5.6 months after COVID-19 diagnosis (range 1–12 months)	121	Median 9 (IQR 10.8–17.9) range 0–18 years	54%	27 (22%) hospitalized	37%
Fink, T. et al., 2021 [27]	Brazil	Prospective observational	Median 4.4 (IQR 0.8–10.7) months after initial COVID-19 diagnosis	53	Median 15 range 8–18 years	42%	18 (34%) hospitalized 4 (22%) ICU	23%
Funk, A. et al., 2022 [28]	United Sates, Costa Rica, Canada, Spain and other	Prospective cohort ^¥^	≥3 months (range 90–120 days) after ED visit	1884	Median 3 (IQR 0–10) range 0–18	53%	447 (24%) hospitalized	6%
Kikkenborg Berg, S. et al., 2022 [20]	Denmark	National cross-sectional ^¥^	≥ 3 months after initial COVID-19 diagnosis (range 3–12 months)	6630	Median 17 (IQR 16.5–18.6) range 15–18 years	42%	594 (9%) self-reported	38%
Kikkenborg Berg, S. et al., 2022 [21]	Denmark	National cross-sectional ^¥^	3 months (range 3–12 months)	10977	Median 10 (IQR 6 6–12.8) range 0–14 years	52%	200 (2%) self-reported	29%
Leftin Dobkin, S. et al., 2021 [29]	USA	Retrospective	Mean 3.2 (SD 1.5) months after acute COVID-19 infection (range 1.3–6.7 months)	29	13 (SD 3.9) range 4–19 years	41%	4 (14%) self-reported	100% *
Osmanov, I. et al., 2022 [30]	Russia	Prospective cohort ^¥^	Median 268 (IQR 233–284) days since hospital admission	518	10 (IQR 3–15.2), range 0–18 years	48%	518 (100%) hospitalized	25%
Ozturk, G. et al., 2022 [31]	Germany	Retrospective	3 months after hospital discharge	50	Median 15 range 5–18 years	56%	10 (20%) reported	28%
Radtke, T. et al., 2021 [32]	Switzerland	Longitudinal cohort ^¥^	≥ 12 weeks after positive COVID-19 test	1355	Median 11 (range 6–16)	47%	NR	4%
Say, D. et al., 2021 [33]	Australia	Cross-sectional	3–6 months after COVID-19 diagnosis	171	Median 3 (IQR 1–8) range 0–18 years	53%	1 (1%) reported	8%
Stephenson, T. et al., 2022 [34]	England	National cohort ^¥^	Median 14.9 (IQR 13.1–19.9) weeks	3065	Range 11–17	37%	NA	66%

COVID-19 = coronavirus disease 2019; SARS-CoV-2 = severe acute respiratory syndrome coronavirus 2; ED = Emergency Department. IQR = Interquartile range; SD = standard deviation; CI = confidence interval. NR = Not reported. NA = Not applicable. ICU = Intensive Care Unit. Long COVID-19 ≥1 persistent symptom or structural change at follow-up. ^¥^ Data collected using a survey, interview or questionnaire, otherwise clinical data was used (e.g., medical histories, physical examinations, lung function testing, chest CT or X-ray, etc.) * Inclusion criteria.

**Table 2:** Pooled prevalence of COVID symptoms in children and adolescents between 3- and 12-months post- infection.

Clinical Manifestations	Studies	Cases	Sample Size	I^2^	Prevalence % (95% CI)
CT or X-ray changes	4	21	185	73	10 (1–19)
Abnormal PFT	3	39	149	90	24 (4–43)
Obstructive pattern	3	11	139	0	7 (3–12)
Impaired diffusion pattern	3	8	129	42	5 (0–10)
Chest pain/tightness	11	374	11,135	100	6 (3–8)
Heart rhythm disturbances/Palpitations	8	344	15,414	98	6 (4–7)
Dyspnea/breathing difficulties	13	1207	22,084	99	16 (14–19)
Persistent cough	15	651	22,708	98	4 (3–5)
Sore throat	4	676	19,596	99	10 (8–13)
Rhinorrhea	4	28	2596	83	2 (0–4)
Persistent fatigue	14	3182	22,721	100	24 (20–27)
Decreased exercise capacity	3	24	126	88	20 (4–37)
Functional limitations (activities of daily living)	2	85	179	91	48 (25–70)

I^2^ index to describe the percentage of variation caused by inconsistency rather than chance between studies in the pooled analyses. Low, moderate, and considerable heterogeneity were indicated by I^2^ ≤ 30%, between 30–50%, and ≥ 75%, respectively.

## Supplementary Materials

The following supporting information can be downloaded at: https://www.mdpi.com/article/10.3390/healthcare10122492/s1, Table S1: Search strategy; Table S2; Quality assessment tool; Table S3: Prevalence of pulmonary function abnormalities, and CT/X-ray abnormalities; Table S4: Prevalence of cardiorespiratory symptoms and fatigue; Table S5: Prevalence of decreased exercise and limitation in daily function.
