# Supplementary material for: Long-Term Effect of COVID-19 on Lung Imaging and Function, Cardiorespiratory Symptoms, Fatigue, Exercise Capacity, and Functional Capacity in Children and Adolescents: A Systematic Review and Meta-Analysis
Source: Healthcare (Basel). 2022 Dec 9;10(12):2492. doi: 10.3390/healthcare10122492 (PMC9778658; doi:10.3390/healthcare10122492)
Supplement: Supplementary file 1 [file healthcare-10-02492-s001.zip › healthcare-2058317-supplementary.pdf]

Supplementary material

Table S1. Search strategy of the systemic review

Ovid MEDLINE(R) and Epub Ahead of Print, In-Process, In-Data-Review & Other Non-Indexed Citations and Daily

**1**	exp Coronavirus/ and (long* or post* or extended or post-acute or persistent or chronic or "long haul*").ti,ab.
**2**	exp Coronavirus Infections/ and (long* or post* or extended or post-acute or persistent or chronic or "long haul*").ti,ab.
**3**	((long* or post* or extended or post-acute or persistent or chronic or "long haul*") adj5 (coronavirus* or corona virus* or OC43 or NL63 or 229E or HKU1 or HCoV* or ncov* or covid* or sars-cov* or sarscov* or Sars-coronavirus* or Severe Acute Respiratory Syndrome Coronavirus*)).tw,kf.
**4**	(or/1-3) and ((20191* or 202*).dp. or 20190101:20301231.ep.)
**5**	4 not (SARS or SARS-CoV or MERS or MERS-CoV or Middle East respiratory syndrome or camel* or dromedar* or equine or coronary or coronal or covidence* or covidien or influenza virus or HIV or bovine or calves or TGEV or feline or porcine or BCoV or PED or PEDV or PDCoV or FIPV or FCoV or SADS-CoV or canine or CCov or zoonotic or avian influenza or H1N1 or H5N1 or H5N6 or IBV or murine corona*).mp.
**6**	(((pneumonia or covid* or coronavirus* or corona virus* or ncov* or 2019-ncov or sars*).mp. or exp pneumonia/) and Wuhan.mp.) adj5 (long* or post* or extended or post-acute or persistent or chronic or "long haul*").ti,ab. [mp=title, book title, abstract, original title, name of substance word, subject heading word, floating sub-heading word, keyword heading word, organism supplementary concept word, protocol supplementary concept word, rare disease supplementary concept word, unique identifier, synonyms]
**7**	((2019-ncov or ncov19 or ncov-19 or 2019-novel CoV or sars-cov2 or sars-cov-2 or sarscov2 or sarscov-2 or Sarscoronavirus2 or Sars-coronavirus-2 or SARS-like coronavirus* or coronavirus-19 or covid19 or covid-19 or covid 2019 or ((novel or new or nouveau) adj2 (CoV or nCoV or covid or coronavirus* or corona virus or Pandemi*2)) or ((covid or covid19 or covid-19) and pandemic*2) or (coronavirus* and pneumonia)) adj5 (long* or post* or extended or post-acute or persistent or chronic or "long haul*")).ti,ab.
**8**	(COVID-19.rx,px,ox. or severe acute respiratory syndrome coronavirus 2.os.) and (long* or post* or extended or post-acute or persistent or chronic or "long haul*").ti,ab.
**9**	("32240632" or "32236488" or "32268021" or "32267941" or "32169616" or "32267649" or "32267499" or "32267344" or "32248853" or "32246156" or "32243118" or "32240583" or "32237674" or "32234725" or "32173381" or "32227595" or "32185863" or "32221979" or "32213260" or "32205350" or "32202721" or "32197097" or "32196032" or "32188729" or "32176889" or "32088947" or "32277065" or "32273472" or "32273444" or "32145185" or "31917786" or "32267384" or "32265186" or "32253187" or "32265567" or "32231286" or "32105468" or "32179788" or "32152361" or "32152148" or "32140676" or "32053580" or "32029604" or "32127714" or "32047315" or "32020111" or "32267950" or "32249952" or "32172715").ui. and (long* or post* or extended or post-acute or persistent or chronic or "long haul*").ti,ab.
**10**	or/6-9

**11**	5 or 10
**12**	adolescent/ or exp child/
**13**	(infant* or child?? or children or teens or teenager* or adolescen*).tw,kf.
**14**	12 or 13
**15**	11 and 14
**16**	(pulmonary or bronch* or cardiorespiratory or cardio-respiratory or respiratory or lung* or breath*).mp.
**17**	15 and 16

Table S2. Quality Assessment Tool for Observational Cohort and Cross-Sectional Studies (n=17)

Author	Year	1	2	3	4	5	6	7	8	9	10	11	12	13	14	Total Score	Quality Rating
Asadi-Pooya, A. [18]	2021	Y	Y	N	Y	N	N	Y	Y	Y	N	Y	N	NA	N	7/14 (50%)	Fair
Ashkenazi-Hoffnung, L. [19]	2021	Y	Y	N	Y	N	Y	Y	Y	Y	N	Y	N	NA	Y	9/14 (64%)	Fair
Brackel, H. [22]	2021	Y	Y	Y	Y	N	N	Y	N	Y	N	Y	N	NA	N	7/14 (50%)	Fair
Buonsenso, D. [23]	2021	N	Y	Y	Y	N	N	Y	Y	Y	N	Y	N	N	N	7/14 (50%)	Fair
Buonsenso, D. [24]	2022	Y	Y	N	Y	N	N	Y	Y	Y	Y	Y	N	N	N	8/14 (57%)	Fair
Dolezalova, K. [25]	2022	Y	Y	Y	Y	N	Y	Y	Y	N	Y	N	N	Y	N	9/14 (64%)	Fair
Erol, N. [26]	2021	Y	Y	N	Y	N	Y	Y	Y	Y	Y	N	N	NA	Y	9/14 (64%)	Fair
Fink, T. [27]	2021	Y	Y	Y	Y	N	Y	Y	Y	Y	N	Y	N	NA	N	9/14 (64%)	Fair
Funk, A [28]	2022	Y	Y	Y	Y	Y	Y	Y	Y	N	Y	N	Y	NA	N	10/14 (71%)	Fair
Kikkenborg Berg, S. [20]	2022a	Y	Y	Y	Y	Y	Y	Y	Y	Y	Y	Y	N	N	Y	12/14 (86%)	Good
Kikkenborg Berg, S. [21]	2022b	Y	Y	N	Y	N	Y	Y	Y	Y	Y	Y	N	NA	N	9/14 (57%)	Fair
Leftin Dobkin, S. [29]	2021	Y	Y	N	Y	N	Y	Y	N	Y	N	Y	N	NA	N	7/14 (50%)	Fair
Osmanov, L. [30]	2022	N	Y	Y	Y	N	Y	Y	N	N	Y	N	N	NA	N	6/14 (43%)	Poor
Ozturk, G. [31]	2022	Y	Y	Y	Y	N	N	Y	Y	Y	N	Y	N	NA	N	8/14 (57%)	Fair
Radtke, T. [32]	2021	Y	Y	N	Y	N	N	Y	N	Y	N	Y	N	N	N	6/14 (43%)	Poor
Say, D. [33]	2021	Y	Y	Y	Y	N	Y	Y	N	Y	N	Y	N	NA	N	8/14 (57%)	Fair
Stephenson, T. [34]	2022	N	Y	N	Y	Y	N	Y	N	Y	N	N	N	NA	N	5/14 (36%)	Poor

Quality of included studies was assessed using the National Institutes of Health (NIH) Quality Assessment tool for Observational Cohort and Cross-Sectional Studies (https://www.nhlbi.nih.gov/health-pro/guidelines/in-develop/cardiovascular-risk-reduction/tools/cohort). **1**. Was the research question or objective in this paper clearly stated? **2**. Was the study population clearly specified and defined? **3**. Was the participation rate of eligible persons at least 50%? **4**. Were all the subjects selected or recruited from the same or similar populations (including the same time period)? Were inclusion and exclusion criteria for being in the study prespecified and applied uniformly to all participants? **5**. Was a sample size justification, power description, or variance and effect estimates provided? **6**. For the analyses in this paper, were the exposure(s) of interest measured prior to the outcome(s) being measured? **7**. Was the timeframe sufficient so that one could reasonably expect to see an association between exposure and outcome if it existed? **8**. For exposures that can vary in amount or level, did the study examine different levels of the exposure as related to the outcome (e.g., categories of exposure, or exposure measured as continuous variable)? **9**. Were the exposure measures (independent variables) clearly defined, valid, reliable, and implemented consistently across all study participants? **10**. Was the exposure(s) assessed more than once over time? **11**. Were the outcome measures (dependent variables) clearly defined, valid, reliable, and implemented consistently across all study participants? **12**. Were the outcome assessors blinded to the exposure status of participants? **13**. Was loss to follow-up after baseline 20% or less? **14**. Were key potential confounding variables measured and adjusted statistically for their impact on the relationship between exposure(s) and outcome(s)? **Total Score,** number of yes; **NA**, not applicable; **NR**, not reported, **N**, not present; **Y**, present. **Quality Rating:** Poor <50%, Fair 50-75%, Good 75%

Table S3. Prevalence of pulmonary function abnormalities, CT/X-ray abnormalities ≥3 months post COVID-19						
**Author**	**n**	**CT/X-ray abnormalities % (n)**	**PFT abnormalities % (n)**	**Impaired diffusion pattern % (n)**	**Obstructive pattern % (n)**	**Restrictive pattern % (n)**
Ashkenazi-Hoffnung et al., 2021 [19]	90	13 (12)	45 (27/60)	2 (1/50)	8 (5/60)	NR
Dolezalova et al., 2022 [25]	39	19 (7)	13 (5)	8 (3)	NR	NR
Fink et al., 2021 [27]	48	2 (1)	NR	NR	NR	NR
Leftin Dobkin et al, 2021 [29]	29	13 (1)	NR	NR	10 (3)	0
Ozturk et al., 2022 [31]	50	NR	14 (7)	10 (4/40)	6 (3/50)	2 (1/50)

COVID-19= coronavirus disease 2019. n=population of the study. CT= Computed tomography. CT/X-ray (consolidation, hyperinflation, bronchial wall thickening, GGO's, pleural effusions, basal limited infiltration, perihilar opacities).NR= not reported or reported in a different format. PFT = pulmonary function testing (abnormal spirometry FEV1 < 80% OR FEV1/FVC < 0.8 or abnormal exercise challenge test or positive bronchodilator response ΔFEV1 ≥ 12% or plethysmography RV/TLC > 125%). Restrictive (FVC <80% and TLC <80% predicted OR reduced TLC below 5th percentile of predicted and normal FEV1/FVC). Obstructive (FEV1/FVC <80% predicted OR reduced FEV1/FVC ratio below 5th percentile of predicted). Impaired diffusion (DLCO <70%).

Table S4. Prevalence of cardiorespiratory symptoms and fatigue ≥3 months post COVID-19

Author	n	Heart Rhythm Disturbances /palpitations % (n)	Chest pain/tightness % (n)	Dyspnea % (n)	Cough % (n)	Wheezing % (n)	Rhinorrhea % (n)	Sputum % (n)	Sore Throat % (n)	Fatigue % (n)
Asadi-Pooya et al., 2021 [18]	58	NR	NR	12 (7)	7 (4)	NR	NR	5 (3)	NR	21 (12)
Ashkenazi-Hoffnung et al., 2021 [19]	90	11 (10)	31 (28)	50 (45)	10 (9)	NR	NR	NR	NR	71 (64)
Brackel et al., 2021 [22]	89	18 (16)	35 (31)	55 (49)	1 (1)	NR	NR	NR	NR	87 (77)
Buonsenso et al., 2021 [23]	98	7 (7)	10 (3/30)	NR	4 (4)	NR	11 (11)	NR	NR	13 (13)
Buonsenso et al., 2022 [24]	428	41 (176)	NR	NR	25 (126)	NR	NR	NR	38 (194)	80 (346)
Dolezalova et al., 2022 [25]	39	NR	18 (7)	31 (12)	49 (19)	NR	NR	NR	NR	NR
Erol et al., 2021 [26]	121	NR	4 (5)	3 (4)	1 (1)	NR	NR	NR	NR	1 (1)
Fink et al., 2021 [27]	53	NR	NR	8 (4)	0 (0)	NR	0 (0)	NR	0 (0)	NR
Funk et al., 2022 [28]	1184	NR	NR	26 (311)	77 (917)	10 (122)	75 (893)	3 (35, and apnea)	29 (341)	1 (21)
Kikkenborg Berg et al., 2022a [20]	5106	1 (69)	1 (70)	4 (183)	1 (52)	NR	NR	NR	1 (37)	11 (547)
Kikkenborg Berg et al., 2022b [21]	10997	<1 (61)	NR	<1 (133)	3 (293)	NR	NR	NR	1 (154)	8 (841)
Leftin Dobkin et al., 2021 [29]	29	NR	NR	97 (28)	52(15)	NR	NR	NR	NR	14 (4)
Osmanov et al., 2022 [30]	518	1 (5/471)	<1 (3/487)	1 (7/503)	1 (5/503)	NR	2 (10/505)	NR	NR	11 (53/496)
Ozturk et al., 2022 [31]	50	NR	10 (5)	10 (5)	6 (3)	NR	NR	NR	NR	NR
Radtke et al., 2021 [32]	109	NR	1 (1/109)	NR	NR	NR	1 (1)	NR	NR	3 (3)
Say et al., 2021 [33]	151	NR	NR	NR	5 (7)	NR	NR	NR	NR	3 (4)
Stephenson et al., 2022 [34]	3065	NR	7 (216)	23 (717)	3 (98)	NR	NR	NR	10 (291)	39 (1196)

COVID-19= coronavirus disease 2019. n=population of the study. NR= not reported or reported in a different format.

Table S5. Prevalence of decreased exercise and limitations in daily function ≥3 months post COVID-19 (number of studies=6)

Author	n	Decreased exercise capacity % (n)	Limitations in daily function* % (n)
Asadi-Pooya et al., 2021 [18]	58	12 (7)	NR
Ashkenazi-Hoffnung et al., 2021 [19]	90	NR	59(53)
Brackel et al., 2021 [22]	89	NR	36 (32)
Buonsenso et al., 2022 [24]	428	NR	NR
Dolezalova et al., 2022[25]	39	8 (3)	NR
Leftin Dobkin et al., 2021 [29]	29	48 (14)	NR

COVID-19= coronavirus disease 2019. n= population of the study. NR= not reported or reported in a different format. *Includes inability, or limited ability to go to school, and an interview assessment performed by a senior pediatrician with a minimum of 10 years of experience to evaluate the effect of COVID on a patient's daily activities.
